# Carney’s triad in an adult male from a tertiary care center in India: a case report

**DOI:** 10.1186/s13256-021-03149-x

**Published:** 2021-11-16

**Authors:** Ghazal Tansir, Nihar Ranjan Dash, Saurabh Galodha, Prasenjit Das, Shamim Ahmed Shamim, Sameer Rastogi

**Affiliations:** 1 Department of Medical Oncology, BRA IRCH, All India Institute of Medical Sciences, New Delhi, India; 2 Department of Gastrointestinal Surgery, All India Institute of Medical Sciences, New Delhi, India; 3 Department of Pathology, All India Institute of Medical Sciences, New Delhi, India; 4 Department of Nuclear Medicine, All India Institute of Medical Sciences, New Delhi, India; 5 Sarcoma Medical Oncology Clinic, BRA IRCH, All India Institute of Medical Sciences, New Delhi, India

**Keywords:** Wild-type GIST, Chondroma, Adrenal tumor, Gastrectomy

## Abstract

**Background:**

Carney’s triad is a rare syndrome comprising gastrointestinal stromal tumor, extra-adrenal paraganglioma, and pulmonary chondroma along with newer additions of adrenal adenoma and esophageal leiomyoma. The triad is completely manifest in only 25–30% cases, with most patients presenting with two out of three parts of the syndrome. Wild-type succinate-dehydrogenase-deficient gastric gastrointestinal stromal tumor forms the most common component of Carney’s triad and is usually multicentric and multifocal. It usually demonstrates indolent behavior and resistance to imatinib; hence, the management remains predominantly surgical. Pulmonary chondromas are commonly unilateral and multiple with slow-growing nature, which allows for conservative management. Adrenocortical adenomas are found in 20% of patients and are usually detected as incidentalomas.

**Case presentation:**

A 49-year-old Asian male presented with upper gastrointestinal bleed and was diagnosed with multiple gastric succinate-dehydrogenase-deficient gastrointestinal stromal tumors. On evaluation, he was found to have left pulmonary chondroma and non-secretory adrenal adenoma, thus completing the Carney’s triad. He underwent surgery with sleeve gastrectomy and excision of the antral tumor nodule, while the adrenal and pulmonary tumors have been under close follow-up.

**Conclusion:**

Literature regarding Carney’s triad is scarce, especially from the Indian setting. Our report aims to highlight the various manifestations of this syndrome with emphasis on management of wild-type succinate-dehydrogenase-deficient gastrointestinal stromal tumor. Radical gastric surgeries do not offer a survival advantage in this condition; hence, more conservative modalities of resection can be adopted.

## Introduction

Carney’s triad was first described in 1977 and was defined as an association of three tumors: gastric epithelioid leiomyosarcoma [later recognized as gastrointestinal stromal tumor (GIST)], extra-adrenal paraganglioma, and pulmonary chondroma [1]. Subsequently, two other tumors were added to this syndrome: adrenal adenoma and esophageal leiomyoma [2].

The GIST that occurs in Carney’s triad is distinct from sporadic GIST in clinical, pathological, and behavioral aspects as described by Zhang *et al.* [3]. GIST in Carney’s triad is found predominantly in young females in the early third decade. The most common primary site is the stomach, and the most common site in the gastric region is the antrum. They are multicentric and multifocal, and have a propensity for metastasis to lymph nodes. Pathologically, they are mostly with epithelioid morphology. They are typically negative in mutation testing for *cKIT* and *PDGFRA* but are succinate dehydrogenase (*SDH*) deficient. Behaviorally, these tumors are slow-growing and are usually resistant to imatinib [3]. In a series by Carney *et al.*, 19 patients received imatinib and partial response was seen only in one patient [4]. The prognosis of these tumors does not depend on conventional factors like tumor size and mitotic count [4]. Even after the diagnosis of metastasis, the outcome remains unpredictable, with many patients showing indolent nature.

Most patients present with two of three symptoms, characterized as incomplete Carney’s triad. Only 25–30% of patients will have fully expressed triad with all three tumors. In a series of 72 patients, the most common presenting tumor was GIST (75%), followed by pulmonary chondroma (15%) and paraganglioma (10%) [2].

The typical treatment of this syndrome is surgical resection of the GIST lesion in a nonmetastatic setting. Most of the GIST tumors in this condition have an indolent course and, usually, surgery of the pulmonary chondroma is not required.

The genes encoding *SDH* subunits have been found to harbor multiple CpG islands in the promoter and exonic regions. Aberrant hypermethylation of these islands encoding the gene loci of SDHB and SDHC subunits has been found by Haller *et al.*, suggesting a role of epigenetic mechanisms in disease pathogenesis [5]. In a study analyzing the genetics of patients with Carney’s triad, Matyakhina *et al.* reported no germline mutations in *SDHB*, *SDHC*, *SDHD*, *c-KIT*, and *PDGFRA* genes. The most significant finding in this study was 1q12-q21 deletion, a region that contains the *SDHC* gene by comparative genomic hybridization (CGH) [6]. However, in another study, Boikos *et al.* found that less than 10% of Carney triad patients had germline mutations of the *SDHx* gene and affected protein function [7].

We hereby describe a case of Carney’s triad who presented to our center with GIST and was subsequently diagnosed with this syndrome.

## Case discussion

**Fig. 1:**
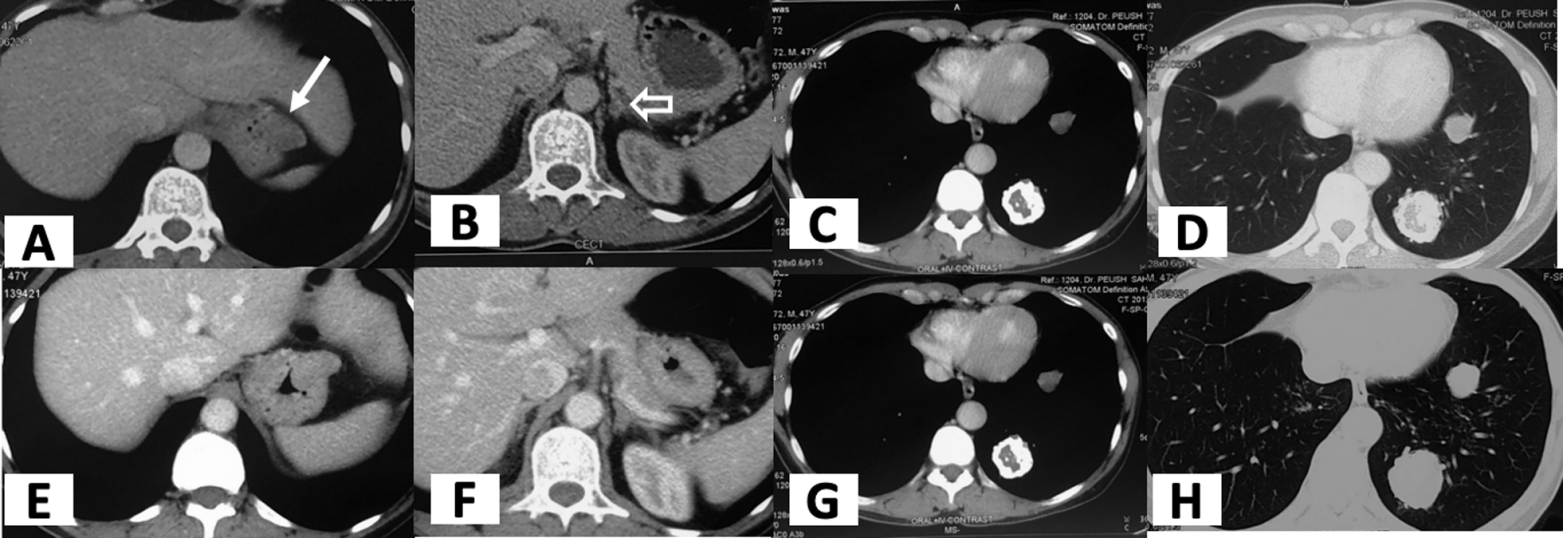
Axial CT showing exophytic gastric fundus mass (marked by arrow) (**A**), left adrenal nodule (marked by arrow) (**B**), and pulmonary calcified nodules/fibroma (**C**,** D**). On repeat scan, no significant change seen in gastric mass (**E**) and lung nodules (**G**,** H**), and left adrenal nodule has slightly reduced in size (**F**)

**Fig. 2:**
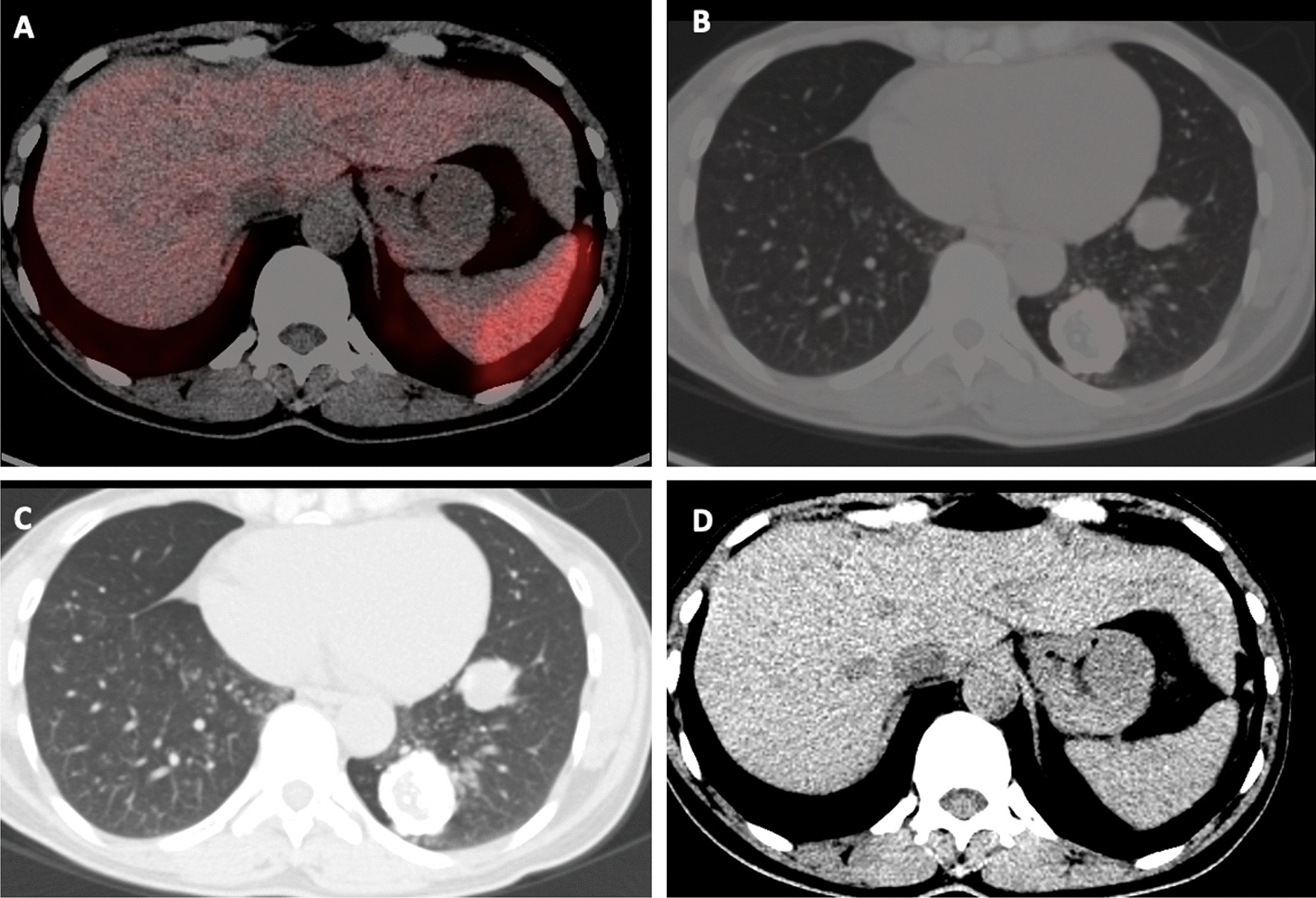
**A** Axial CT section abdomen showing endo-exophytic lesion from gastric fundus showing no significant tracer uptake on fused PET-CT (**B**).** C** Axial CT thorax showing soft tissue density nodular lesions in left lung lower lobe with no significant tracer uptake on fused PET-CT (**D**)

**Fig. 3:**
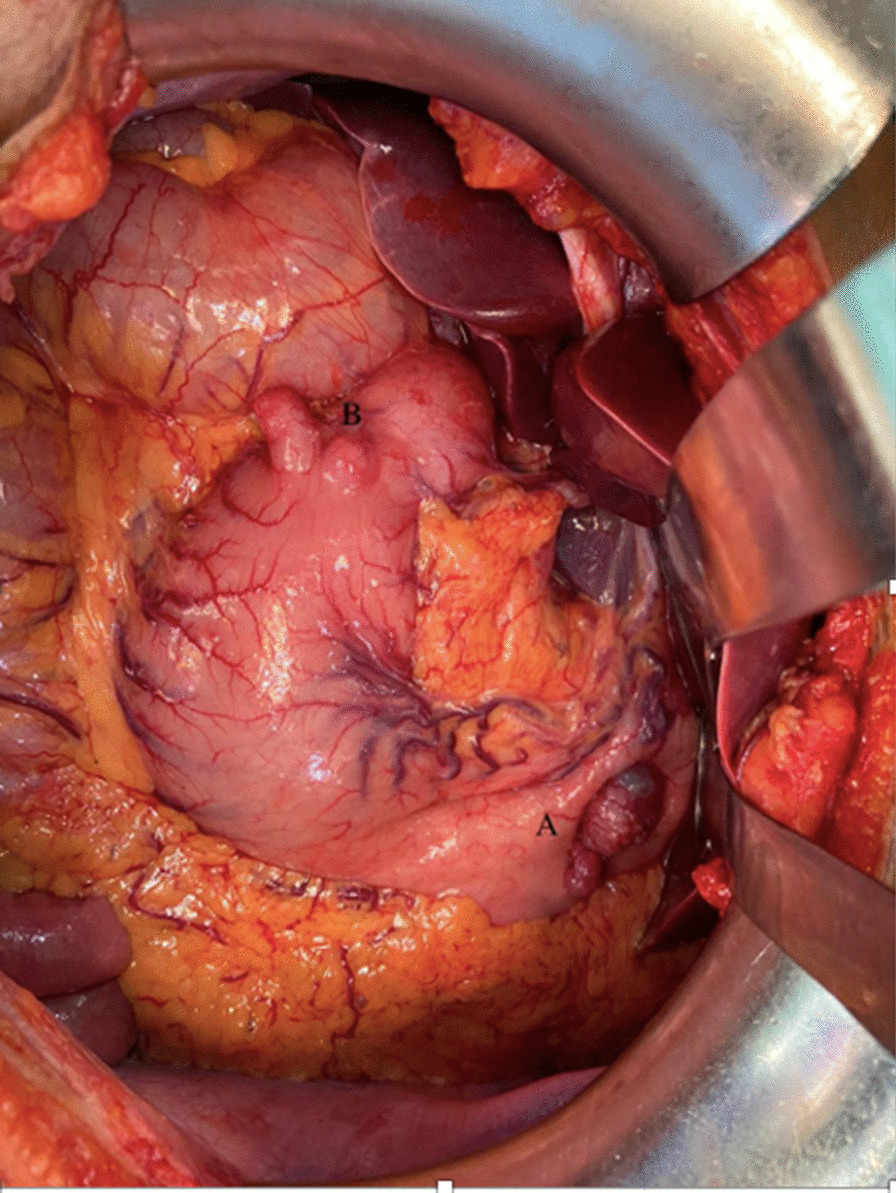
Intraoperative findings of multiple fundal (**A**) and single antral (**B**) tumor nodules

**Fig. 4:**
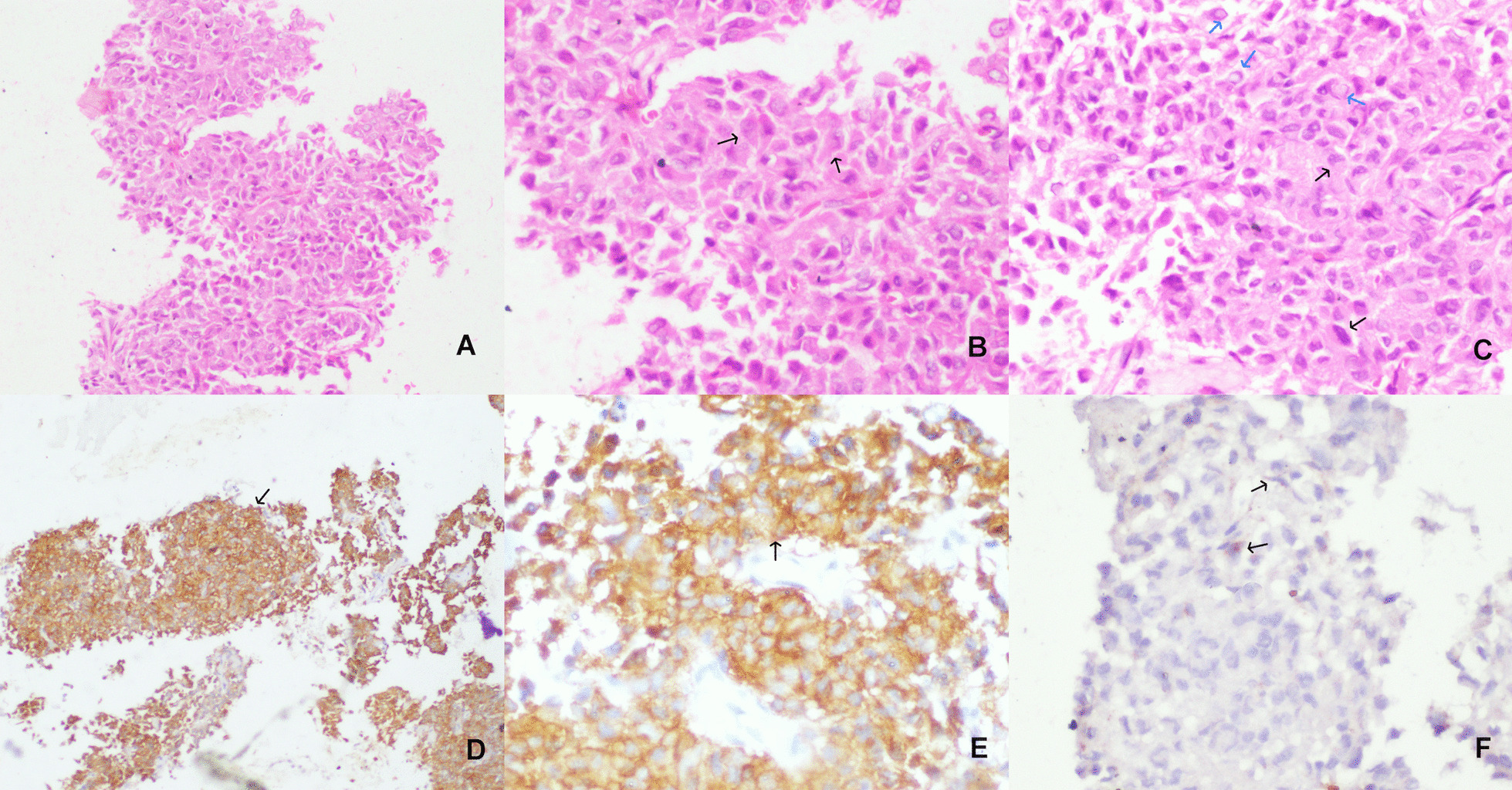
Photomicrographs showing a GIST, predominantly composed of pleomorphic epithelioid cells (marked by arrows) with moderate amount of eosinophilic cytoplasm, occasional nuclear pseudoinclusions (**B**), and signet ring type of tumor cells (blue arrows,** C**) (**A** ×100,** B** and** C** ×200). The tumor cells show membranous and cytoplasmic positivity (arrows) for CD117 (**D** ×100) and DOG-1 (**E** ×200) stains (marked by arrow). The tumor was negative for SDH B stain, while the stain is positive in vascular endothelial cells (marked by arrows), used as an internal control (**F** ×200)

**Fig. 5:**
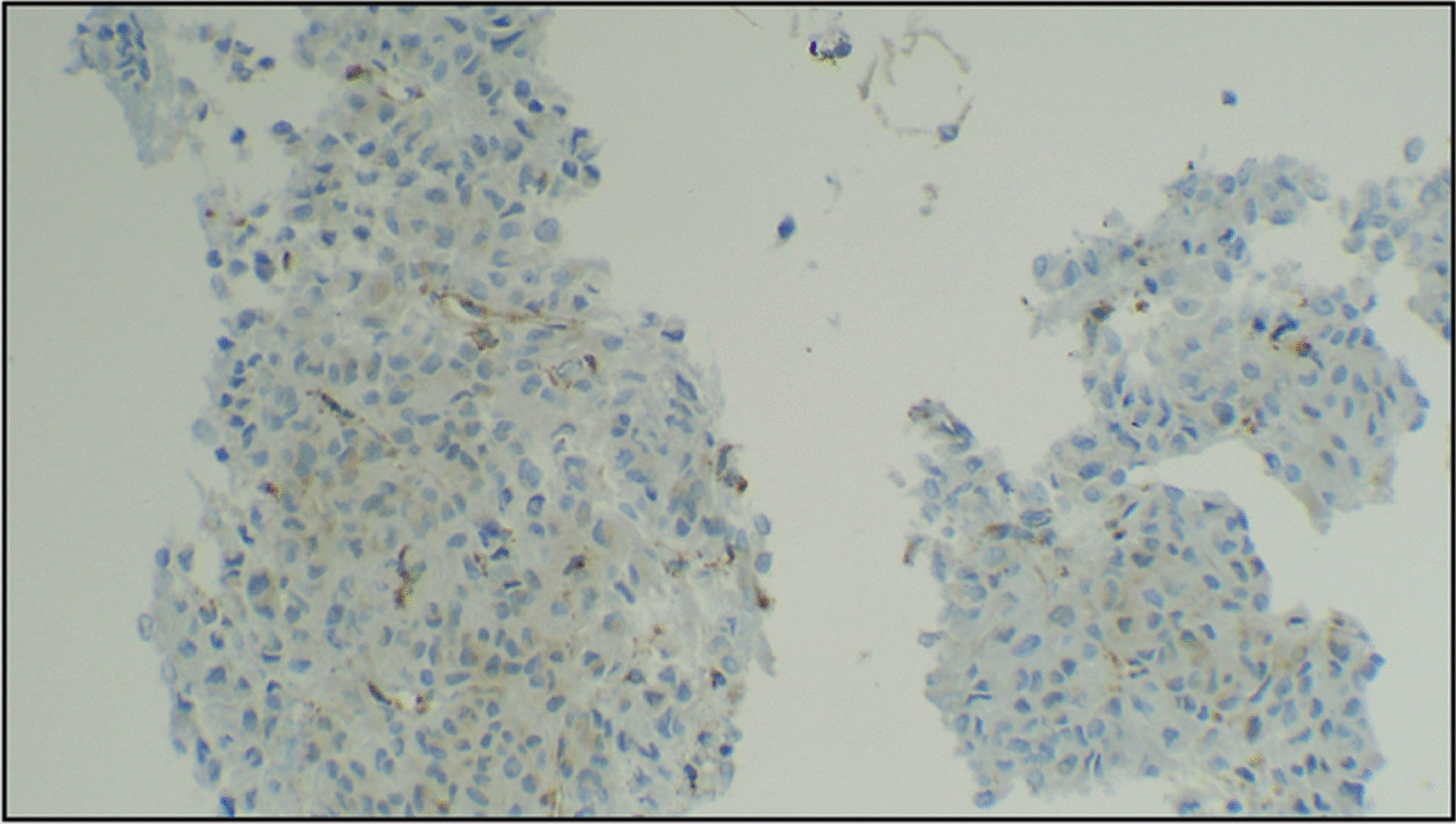
The expression of SDH-B is deficient in the tumor cells, while the expression is retained as cytoplasmic granular immunostaining in endothelial cells serving as internal control

The patient is a 49-year old Asian male who presented to us with complaints of pain abdomen, hematemesis, and melena (Table 1). He underwent an endoscopic evaluation that revealed multiple gastric antral and fundal nodules, and biopsy from the antral nodule was suggestive of GIST. The patient was evaluated by contrast-enhanced computed tomography (CECT) of the abdomen, which revealed two well-circumscribed mildly enhancing exophytic masses involving proximal fundus measuring 3.4 × 2 cm and 10 × 8 cm in size and another single lesion involving the pylorus (Fig. 1). Moreover, the lower lobe of the left lung showed three well-defined soft tissue lesions with dense and coarse calcifications along with multiple bilateral peribronchovascular nodules. A radiological impression of multiple gastric GISTs along with left pulmonary chondromas with a background of infective infiltrates was made. Keeping the possibility of Carney’s triad, a gallium [DOTA,1-Nal^3^]-octreotide (DOTANOC) scan was performed, which a revealed hypodense nodule of size 9 × 8 mm in the body of the left adrenal gland, suggestive of an adrenal adenoma (Fig. 2). The adrenal adenoma was non-secretory, with absence of hypertension or Cushingoid features and no biochemical evidence of urinary and serum cortisol or catecholamine excess. Intraoperative findings in the stomach were two antral and three fundal nodules for which sleeve gastrectomy and wide excision of the antral nodules was done (Fig. 3). The rest of the bowel was found free of tumor nodules. The adrenal adenoma has been kept under observation because of spontaneous reduction in size and non-secretory nature. Histopathology of the tumor specimen yielded findings of tumor tissue with epithelioid cells morphologically and cluster of differentiation (CD) 117 and Discovered on GIST-1 (DOG-1) were strongly positive (Fig. 4). *SDH B* was found deficient on immunohistochemistry (IHC) (Fig. 5). The patient is doing well after surgery and is now on regular follow-up by imaging and endoscopic evaluation.

**Table 1:** Salient timeline events during the clinical course, evaluation, and management of the patient

Timeline of events	Clinical course
*T* = 0	Patient presents with pain abdomen, hematemesis, melena for 4 months
*T* = 1 month	CECT chest and abdomen: Presence of well-circumscribed exophytic and endophytic gastric fundal masses with well-defined soft tissue lesions containing coarse calcifications in left lower lobe of lung
*T* = 3 months	Gallium-DOTANOC scan: soft tissue lesions in left lower lobe of lung with dense peripheral calcifications and no significant uptake, 3.5 × 3.4 cm, suggestive of chondromas. There was presence of exophytic multiple gastric lesions and left adrenal nodule No evidence of catecholamine excess in serum or urinalysis
*T* = 4 months	Patient underwent sleeve gastrectomy for removal of gastric fundal nodules, along with wide excision of the antral nodules

## Discussion

Carneys triad consists of a unique subset of wild-type GIST, and it is rarely documented from the developing world [8]. Ours is a unique case because 85–90% of the patients reported with Carney’s triad are females [2, 4]. A case series by Carney described 77 patients with a median age of 20 (range 7–48 years), with most GIST patients having bleeding as the initial presentation due to mucosal ulceration [2]. The age of our patient lies on the higher side of the spectrum, with onset at 48 years of age, and his first presentation with gastrointestinal bleed is similar to the description in previous literature. In a case series by Zhang *et al.* [3] describing 104 patients of Carney’s triad, the majority of GIST tumors were multiple and antral-based (61%) and had epithelioid morphology with cKIT positivity, as in our case. IHC testing in an accredited laboratory revealed SDH B deficiency together with positive internal control, establishing the diagnosis of wild-type GIST.

Our patient underwent stomach conservation surgery as per the available data. In a study on a large cohort of wild-type GIST patients, from the National Institute of Health (NIH) Pediatric and Wildtype GIST Clinic, surgical factors like microscopic margins and type of resection were not associated with a difference in event-free survival (EFS) [9]. This was the basis on which we could obviate total gastrectomy in our patient.

Pulmonary chondromas are found in 75% of patients and can be single, unilateral and multiple, or bilateral in decreasing order of frequency. Forty percent of patients have been found to have multiple unilateral pulmonary chondromas [2]. The lesions are usually asymptomatic and detected during the course of evaluation for GIST. Though their natural history remains to be determined, they are usually slow-growing and do not impair lung function and, hence, can only be observed. Our patient also has unilateral multiple chondromas, which we chose not to biopsy because of typical radiological appearance and have kept under observation because of asymptomatic disease.

Adrenal adenomas can be found in 20% of patients of Carney’s triad, and can be unilateral or bilateral with a size ranging from 0.4 to 4 cm [2]. Our patient also had an asymptomatic, unilateral, non-secretory adrenal adenoma 0.9 × 0.8 cm in dimension. In a study by Carney *et al.* on 149 patients of Carney’s triad, 28 had adrenocortical tumors [10]. Adrenal asymptomatic neoplasm was usually a late finding, and the majority were unilateral as in our study. Four out of 28 patients had bilateral disease, and only one adrenocortical tumor was found to be functional. Surgical resection had been performed in 50% of cases and none recurred after resection, while the rest were placed under observation only. Based on results from this study, we also avoided surgical resection of the adrenal adenoma and are actively following up the lesion by imaging and biochemical assays. Though the prevalence of paragangliomas in Carney’s triad is 10% [2], we did not find any evidence of paraganglioma in our patient after thorough workup.

Post-surgery, we have not given adjuvant imatinib in this case, as supported by the latest guidelines for the treatment of wild-type GIST [11]. The patient has undergone genetic counseling and needs some time to consider proceeding with germline mutation testing.

There is only a single case report on Carney’s triad from India [11], and this lacuna in literature could stem from a lack of dedicated wild-type GIST clinics, lack of wide availability of SDH B immunohistochemistry, unawareness about the importance of routine mutation testing, and less prevalent knowledge about such rare diseases [12]. We suggest more prospective and collaborative studies focusing on GIST and uncommon conditions such as Carney’s triad to bridge this prevalent knowledge gap.

## Conclusion

This is a rare case of gastric GIST that highlights the various manifestations of Carney’s triad and underscores the importance of SDH examination in wild-type GIST. It is important to include routine mutation testing in cases of GIST, and this practice must be brought to the fore in the Indian setting as well. Better awareness and larger-scale studies will help create more knowledge about such rare syndromes and aid in diagnosis and patient management.

## Data Availability

All data generated are included in the published article manuscript.

## Abbreviations

GIST: Gastrointestinal stromal tumor
SDH: Succinate dehydrogenase
